# Supplementary material for: Iterative improvement in the automatic modular design of robot swarms
Source: PeerJ Comput Sci. 2020 Dec 7;6:e322. doi: 10.7717/peerj-cs.322 (PMC7924708; doi:10.7717/peerj-cs.322)
Supplement: Supplemental Information 3 [file peerj-cs-06-322-s003.zip › argos3/doc/api/standalone/a00401_source.html]

ARGoS: core/utility/plugins/vtable.h Source File


- Main Page
- Related Pages
- Namespaces
- Classes
- Files

- File List
- File Members

# core/utility/plugins/vtable.h

Go to the documentation of this file.

```
00001 
00081 #ifndef VTABLE_H
00082 #define VTABLE_H
00083 
00084 #include <vector>
00085 #include <cstddef>
00086 
00087 #include <argos3/core/utility/logging/argos_log.h>
00088 
00089 namespace argos {
00090 
00094    template <typename BASE>
00095    struct STagCounter {
00096       static size_t Count;
00097    };
00101    template <typename BASE>
00102    size_t STagCounter<BASE>::Count(0);
00103 
00107    template <typename DERIVED, typename BASE>
00108    struct STagHolder {
00109       static size_t Tag;
00110    };
00111 
00115    template <typename DERIVED, typename BASE>
00116    size_t GetTag() {
00117       size_t& unTag = STagHolder<const DERIVED, const BASE>::Tag;
00118       if(unTag == 0) {
00119          /* First call of this function, generate non-zero tag */
00120          ++STagCounter<const BASE>::Count;
00121          unTag = STagCounter<const BASE>::Count;
00122       }
00123       return unTag;
00124    }
00125 
00129    template <typename DERIVED, typename BASE>
00130    size_t STagHolder<DERIVED, BASE>::Tag = GetTag<DERIVED, BASE>();
00131 
00135    template <typename BASE>
00136    struct EnableVTableFor {
00137       template <typename DERIVED>
00138       size_t GetTagHelper(const DERIVED*) const {
00139          return GetTag<DERIVED, BASE>();
00140       }
00141    };
00142 
00146 #define ENABLE_VTABLE()                         \
00147    virtual size_t GetTag() {                    \
00148       return GetTagHelper(this);                \
00149    }
00150 
00154    template <typename CONTEXT, typename BASE, typename FUNCTION>
00155    class CVTable {
00156    public:
00157       template <typename DERIVED>
00158       void Add(FUNCTION t_function) {
00159          /* Find the slot */
00160          size_t unIndex = GetTag<DERIVED, BASE>();
00161          /* Does the table have a slot for this index? */
00162          if(unIndex >= m_vecVTable.size()) {
00163             /* No, new slots must be created
00164              * Fill the slots with the default function that handles BASE
00165              * or NULL if such function does not exist
00166              */
00167             /* Get the tag associated to the base class */
00168             const size_t unBaseTag = GetTag<BASE, BASE>();
00169             FUNCTION tDefaultFunction = 0;
00170             if(unBaseTag < m_vecVTable.size()) {
00171                tDefaultFunction = m_vecVTable[unBaseTag];
00172             }
00173             /* Create new slots up to index+1 and fill them with tDefaultFunction */
00174             m_vecVTable.resize(unIndex+1, tDefaultFunction);
00175          }
00176          m_vecVTable[unIndex] = t_function;
00177       }
00178 
00179       FUNCTION operator[](size_t un_index) const {
00180          if(un_index >= m_vecVTable.size()) {
00181             un_index = GetTag<BASE, BASE>();
00182          }
00183          return m_vecVTable[un_index];
00184       }
00185 
00186       inline size_t Size() const {
00187          return m_vecVTable.size();
00188       }
00189 
00190    private:
00191       std::vector<FUNCTION> m_vecVTable;
00192 
00193    };
00194 
00198    template <typename CONTEXT, typename BASE, typename FUNCTION>
00199    CVTable<CONTEXT, BASE, FUNCTION>& GetVTable() {
00200       static CVTable<CONTEXT, BASE, FUNCTION> cVTable;
00201       return cVTable;
00202    }
00203    
00204 #define INIT_VTABLE_FOR(BASE)                   \
00205    struct SVTableInitializerFor ## BASE {       \
00206       SVTableInitializerFor ## BASE() {         \
00207          GetTag<BASE, BASE>();                  \
00208       }                                         \
00209    } sVTableInitializerFor ## BASE;
00210 
00211 }
00212 
00213 #endif
```

---

Generated on 10 Jul 2018 for ARGoS by 
 1.6.1
